# Rural Public Health Workforce Training and Development: The Performance of an Undergraduate Internship Programme in a Rural Hospital and Healthcare Centre

**DOI:** 10.3390/ijerph16071259

**Published:** 2019-04-09

**Authors:** Luis Miguel Dos Santos

**Affiliations:** Woosong Language Institute, Woosong University, Daejeon 34514, Korea; luisdossantos@woosong.org

**Keywords:** adult learning, andragogy, healthcare management, human resource, internship, performance evaluation, public health, training, rural hospital, rural healthcare

## Abstract

Workforce shortages in the field of public health and healthcare are significant. Due to the limitations of career opportunities and compensation, rural hospitals and healthcare centres usually have on-going career openings for all departments. As a result, university departments of public health and healthcare management, and rural hospitals and health centres may need to establish internship and training programmes for undergraduate senior-year students in order to provide opportunities and human resource opportunities for both students and public health professions. The research examined the performance, feedback, and opinions of a university-based one-year-long on-site internship training programme between a university public health and healthcare undergraduate department and a regional hospital and healthcare centre in a rural region in the United States. Individual interview data were collected from management trainees and focus group activities data were collected from hospital departmental supervisors who have completed this one-year-long on-site internship training programme. The results offered an assessment of performance and evaluation of how a one-year-long internship programme could be beneficial to hospitals and health centres in the areas of human resources, manpower management, and skill training to prospective professionals in rural and regional communities. Also, the study provided a blueprint and alternative for universities and partnered sites to redesign and improve their current internship programmes which may better fit their needs for their actual situations.

## 1. Introduction

Students and professional leaders are increasingly realising that internships and hands-on experience not only offer a means of applying skills but also provide valuable experience that can be beneficial for both the students and the organisations involved. A professional and on-site-based internship is an opportunity for undergraduate students to engage in vocation-oriented experiences, knowledge, and practices within their university curriculum by taking on responsibilities for monitored and planned duties in a practice-based professional setting [1,2]. For those interested in being well-equipped as they enter a professional career in the field of public health and healthcare management, adequate preparation is not simply a matter of graduating from university and stepping into a career [3]. Some experts have argued that the preparation of public health and healthcare professionals should concentrate on the types of skills that are essential for these workers, particularly for university graduates who are seeking managerial positions [4]. As a result, a large number of public health and healthcare faculties and schools have established internships, gap-year partnerships, and on-site internship training for upper-level undergraduate students to learn practical skills before they enter the workforce [5].

Currently, due to the rapid economic and population development occurring in North America, the public health and healthcare profession is searching for skilled professionals who can fill positions from junior levels to executive levels [6], particularly in rural regions where it is more difficult to attract public health and healthcare professionals. In the public health and healthcare profession, it is important for the leadership to make the long-term investments of attracting potentially qualified professionals to be trained as frontline leaders. Considering the array of different positions in each specialised department in regional hospitals and health centres, the process of recruiting, training, and placing appropriately skilled professionals within a short period of time is essential [7]. Therefore, these topics will be of interest for current research projects. Although healthcare is a widely used term in the public health profession, operations management and administrative management are two other major segments of the public health and healthcare profession. In that profession, the most common types of operational divisions are health promotion, disease prevention, population and environment, counselling, and human services. In the administrative divisions, the most common types of departments are epidemiological research, health administration, public communication and marketing, training and development, and finance and accounting, and human resources. These two divisions create and run the daily operations within the healthcare system [7,8,9].

The importance of specialised departments in the public health and healthcare profession imposes a demand for establishing and placing skilled professionals within each individual department and determines what types of specialised training and skills are needed for the various professional workers [10]. To train the potential leaders for each individual department, the public health and healthcare profession can provide on-site training for potential management trainees. One appropriate way to recruit qualified management trainees is to offer an on-site internship training programme that will recruit upper-level undergraduate students who have a strong foundation in public health and healthcare management [11].

A long-term, stable, and effective on-site internship training programme agreement between a university and the public health and healthcare profession can provide the advantage of ensuring undergraduate students’ employment after graduation on the one hand, and on the other hand, the benefit of fulfilling the needs of public health and healthcare leaders to recruit trained and skilled professionals who have the desired foundation in practice and knowledge of organisational structure [4,12,13,14]. These types of training programmes directly contribute to the development of vocation-oriented undergraduate programmes and to the effective growth of the public health and healthcare profession in total. Employees with specialised knowledge in the public health and healthcare profession and the organisational knowledge of a regional hospital and health centre are elements that positively influence performance in the workplace. New employees of a department can take up to a year to adapt to their new workplace environment, and its leadership styles, organisational behaviours, and administrative procedures. Therefore, if upper-level university students can spend their last academic year in that location, the partner regional hospitals and health centres may have a group of trainees that can satisfy the immediate shortage on the one hand, and after their graduation potential supervisors in each department, on the other hand [15].

### 1.1. The Background of the On-Site Internship Training Programme

The current on-site internship training programme is a one-year-long engagement between a university and a partnered regional hospital and health centre in the mid-western rural region in the United States of America. In the United States, an internship refers to a student placement programme for students currently enrolled in and pursuing studies at a degree- or certificate-granting postsecondary academic institution. Although internship programmes could enrol individuals who have completed from similar institutions no more than one year prior to their start date, this study focused on the currently enrolled students’ experience in the designated area. Students who participate in this on-site internship training programme are senior-year undergraduate students majoring in the field of public health and healthcare management. During the senior year of their degree programme, students must participate in this on-site internship training programme, in which they must work at least part-time (i.e., carry at least a half-time workload) at the partnered regional hospital and health centre and must attend lectures after working hours and on weekends. Although the university, students, and partnered regional hospital and health centre may negotiate the work duration and schedule of the trainees, each student’s total working hours per week should not exceed 36 hours. It is worth to note that the 36-hour standard is flexible. During the semester and school terms, students can work and study no more than 36 hours in total. It is recommended that students should reach the 36-hour standard in order to gain the expected achievement from this on-site internship training program. For example, if a student is taking 15 credit-hour in a semester, the additional 21 working hours are recommended for the on-site internship training programme. During the vacation and school breaks without coursework, the students may negotiate with the hospital for scheduling. Therefore, up to 36 working hours are recommended.

Unlike other internships that are one-semester long, the current internship programme requires trainees to intern in all functional departments in the internship regional hospital and health centre for a full calendar year (i.e., 12 months). Each trainee must work in each individual department for approximately two months. Therefore, trainees experience each of the regional hospital and health centre’s functions during the internship.

Due to the unique feature of working in all of the functional departments for two months per term under the one-year-long agreement, the one-year-long on-site internship training programme is designed so that trainees will gain a general perspective of the management at the interned organisation (i.e., the regional hospital and health centre).

It is worthwhile to note that trainees may be required to work during school breaks and national holidays, as a requirement of the agreement. Therefore, due to that requirement, the trainees may work as full-time employees during school breaks and other negotiated periods.

### 1.2. Significance of the Research

The current research examined the performance, feedback, and opinions of a university-based one-year-long on-site internship training programme between a university and a regional hospital and health centre in the mid-western rural region in the United States of America. There are four significant points of this study.

First, human resource and workforce shortages in the fields of public health and healthcare are significant and extreme [16]. Hospitals and health centres located in urban and metropolitan regions may find it easier to attract employees due to their large populations, human resources, numbers of colleges and universities interned students, and economic considerations. However, regional hospitals and health centres usually do not have the advantages as urban hospitals and health centres, such as attractive compensation, and abundant career opportunities. Therefore, regional hospitals and health centres usually have on-going career openings for all departments. As a result, a university and a regional hospital and health centre in the rural United States had established the internship programme to train, and possibly, attract interned university graduates to stay for career opportunities after university graduation.

This study was a performance evaluation of the on-site internship programme between a university and a regional hospital and health centre. This research is important because it serves as a blueprint for rural and regional hospitals and health centres with similar backgrounds to establish effective internship programmes.

Second, a large amount of research and several reports have been conducted on this issue. The findings were mostly reported in a quantitative form, which could neglect the economic backgrounds, human factor consideration, in-depth understanding, and core sharing [17].

Third, most of the previous performance evaluation reports have tended to collect the feedback and opinions from both university supervisors and the leadership of interned sites. The feedback and opinions from the programme trainees and their departmental supervisors have been ignored [18]. Because such internship programmes aim to narrow the gaps between professional needs and employee shortages, it is essential to collect in-depth feedback and opinions from the trainees who have participated in this on-site internship training programme, as well as those of the department supervisor (i.e., mentor) who guided those trainees. These two parties are the groups who have engaged in the programme for one year and who will work together after the completion of the programme at the same workplace. Thus, without their feedback and opinions, there are no ways to improve the current on-site internship training programme [19].

Fourth, a one-year-long on-site internship training programme is not common in current academia, and the current one-year long internship in this study is one of the first programmes that last for more than two semesters (i.e. included the breaks and inter-sessions). No performance evaluations have been reported in this area. Therefore, the results of this research study could answer four significant points about the current talent-management issues in the field of public health and the healthcare profession [20].

Fifth, application to the international context and curriculum reform is one of the priorities of this study. In fact, the current study mainly focused on the applications and problems in the rural region in North America. However, the researcher provided applications and recommendations in the implementation chapter about how the outcomes of this study may be beneficial for health professionals in Europe and Asia. Although the North American practices may not entirely fit the environment and situation in other international locations, sites, and health facilities, the outcomes of this study may provide the blueprints, directions, and samples for potential reforms and developments for health science and public health internship programmes.

### 1.3. Definition of Terms

A gap year refers to a period of time between a quarter to two years in which people take a break from their education or employment, where the break serves as a benefit for career development. Gap year activities are not uncommon in the United Kingdom. However, in North America, gap year activities are not common as most of the students tended to conduct a concurrent internship during their final year of education.

Post-graduation internship refers to a paid or unpaid employment after graduation. In general, undertaking a post-graduation internship is uncommon for an undergraduate students and recent university graduates in North America.

Internship or placement refers to paid, unpaid, or credit-based employment that takes place during the summer but may be completed during the academic year in the North American environment. In this study, the on-site internship is referring to this situation.

## 2. Literature Review 

### 2.1. Theory of Adult Learning: Andragogy

The Theory of Adult Learning, or Andragogy [21,22,23,24,25], served as the underpinning for the conceptual framework of this study. Researchers defined the study of adult learners as having characteristics (i.e., andragogy) that differ from those of the study of younger learners (i.e., pedagogy). Before the introduction of andragogy, research focused on adult learners and adult learning behaviours was ignored [25]. During the last century, as the numbers of returning students and nontraditional students grew significantly, the idea of adult learning was established and evolved into the theory of andragogy.

Andragogy and adult learning behaviours are significant for five reasons (1) individuals have an independent self-concept and idea of who can direct their own learning and planning, (2) individuals have accumulated life experiences that can support their ideas of learning, (3) individuals have learning demands and desires that are related to changing social roles, (4) individuals have the desire to solve problems and an excitement for understanding and applying their newly learnt knowledge in real-world situations, and (5) individuals are self-motivated and can motivate others to learn [21,22,23,24,25]. Based on these characteristics, researchers further narrowed the idea of andragogy into the theory of adult education, the theory of the learning behaviours of adult learners, the theory of the application and technology of adult learning, the learning styles of adults, the technique of adult learning, and a series of assumptions [26].

Unlike pedagogy or the learning methodology of younger learners, adult learners tend to gain knowledge based on their prior knowledge and their needs for personal development. Researchers identified eight factors that can be applied in adult learning planning and programmes [27], as listed in Table 1.

The features of andragogy advocate that the learning curriculum, teaching materials, and expectations for adults should be negotiated between mentors and mentees. When andragogical factors are applied in practice, both mentors and mentees become partners in negotiating the teaching content and materials to meet the students’ desires for learning [28]. The researcher suggested that andragogy is a student-centred methodology in the field of adult learning. The features of andragogy suggest that mentors establish positive engagements with the learners, thereby allowing the sharing and exchange of life experiences [24]. Evaluation styles can be negotiated by all mentors and mentees, in order to achieve the goals of the programme. In fact, adult learners tend to gain knowledge and skills from the engagement of prior experience and new knowledge, and that process can contribute to their needs. The mentors may serve as facilitators, but not as teachers, when they impart knowledge [29,30].

### 2.2. Internships and Learning Effectiveness

Recent reports have indicated that engaging students in vocation-oriented internships and placements are an effective way to enhance their professional skills and employability. Internship experiences help students increase their opportunities for different positions after they graduate from university, regardless of their university majors, but particularly if they graduate with non-vocation-oriented degrees [31,32]. Graduates with internship experience are more likely to be chosen for interviews than those without such experience [33]. In addition, students with internship experience are more likely to be hired by their internship organisation after their university graduation.

The higher potential to be selected for interviews indicates that internships produce significant results that can allow trainees to combine their classroom knowledge and practical work experience. In the field of internship studies, researchers have indicated that trainees can gain knowledge, practical skills, real-world experience, and awareness through their internship experiences with their mentors and that that combination prepares them well for becoming professional workers in their field [33]. In addition, internships at a workplace allow students to gain soft skills from their co-workers—such as communication skills, interactive practices, and team building, all of which are necessary for the field of public health and the healthcare profession [5].

However, recent reports on internship performance in the field of public health and healthcare profession have tended to be quantitative reports that measured performance on the basis of only two factors (1) the feedback from the departmental supervisors, and (2) the feedback from university instructors. An internship performance evaluation indicated satisfaction regarding the performance of a group of interned students, based on evaluations by managers and supervisors from the partnered organisation [34]. Although the managers and supervisors indicated that nearly 60% of their interned students received a passing grade in their evaluation, no further suggestions were given as to how to assist the remaining 40% for further development. Human beings absorb knowledge and behaviours based on the influence of mentors and peers. The internship organisation, as the medium for learning, is involved with both trainees and supervisors. Therefore, the lack of feedback and opinions from trainees cannot reflect a holistic evaluation of the programme’s performance [35]. Another performance evaluation also indicated that students with internship experience could increase their professional skills and marketability in the field. That measurement only involved the feedback and opinions from employers regarding how well the students achieved expectations. However, the reasons why nearly one-third of the students had lower performance were still unknown because the feedback was one way, from the employers [36]. Because no feedback and opinions came from the interned students, no further suggestions and improvements could be established [37]. In sum, internships have the potential to expand the horizons of many individuals, from unskilled students to professional workers. Therefore, these results need to be explored further, in different environments and workplaces.

### 2.3. Gap Year or Participating in a Post-Graduation Internship in the Field of Health Science

Jones [38] refers to a gap year as a period of time between a quarter to two years in which people take a break from their education or employment, where the break serves as a benefit for career development. Gap year activities are not uncommon in the United Kingdom [39]. During the 2000s, nearly 50,000 participants were enrolled in one of the 100 academic programmes with gap year opportunities. Abdullah [40] suggests that a gap year provides opportunities for students to understand their long-term career development and the working environment of their profession. Further, it also allows students to gain soft skills, communication skills, and leadership skills, all of which are required in most professions.

However, some researchers [41,42] indicate that taking a gap year in the field of health science is uncommon, as health and social care professionals tend to be licensed practitioners and staff with trained skills. Although interned students may take opportunities to be staff members at a health science site, these sites tend to recruit concurrent internship candidates who can apply their classroom knowledge to the workplace [43]. Therefore, in North America, a gap year is not a common and popular activity, as most American undergraduate programmes tend to establish internship programmes between the university and professional sites, particularly in the field of science and health science [44]. Although the benefits of taking a gap year in the American education system are thus inapplicable (and therefore lacking), the author will discuss the more applicable benefits of concurrent internships in the following section.

In general, undertaking a post-graduation internship is uncommon during an undergraduate degree and for recent university graduates. North American students tend to conduct concurrent internship programmes during their final year of university study. Similar internship programmes, however, can be found in the Doctor of Psychology programme in the field of clinical psychology, for example. Advanced doctoral degree students (e.g. those who have completed all the required coursework) in the field of clinical psychology are required to complete a doctoral psychology internship programme in order to gain knowledge as entry-level psychology practitioners [45].

### 2.4. Benefits of Undergraduate Internship Programmes

In North American undergraduate programmes, internships usually take place during the summer but may be completed during the academic year. An internship usually involves supervision by experienced mentors both on-site and in the relevant university department. Purposive directions, assignments, jobs, responsibilities, and training are designed for interns, who may receive payment, stipends, or credit toward degree requirements [46]. The idea of concurrent internships and experiential training for undergraduate degree seekers is not new. Although some degree programmes tend to provide purely academic and grammatical studies, vocational courses, health science, management, and teacher-education programmes tend to also have a more experimental and practical focus beyond the traditional classroom environment [47].

Three primary groups are required for internship or placement: students, employers, and university faculty members [48]. For nearly a century, all three parties believed that field experience helps students to become junior professionals in their field. Some researchers [49] assert that internship programmes provide directed learning experiences, for example, to develop strategic managerial skills and communication skills, which cannot be delivered in a classroom.

Particularly in the field of health science, the mentoring process and knowledge exchange from supervisors’ on-site experience at the workplace positively enhance learners’ experience in terms of interpersonal and intrapersonal communication, textbook knowledge and its on-site practical application, and the relationship between patient and health professionals [50]. For more than three decades, research studies have proved that internships are essential for and positively impact health science students. Researchers [51] note that there are both direct and indirect benefits. Researchers show that receiving internship training during their undergraduate voyage allowed students to gain a sense of belonging regarding their professions. In fact, recent university graduates changing their career pathways may cost employers, departmental supervisors, and the student themselves significant resources. Completing concurrent undergraduate internship training during the undergraduate voyage not only assists students in exploring and developing their understanding of their chosen profession, but also reduces turnover rate, releases pressure on human resources, and reduces rates of mismatching. Researchers [52] have also suggested that the hospital-based student internship programmes between the University of Pittsburgh Medical Centre and other 19 community-based hospitals successfully placed a number of future pharmacists into the workplace after the internship programme. The research suggested that pharmacists are not trained as communication experts. The internship programme provided career opportunities for related health professionals in the right positions before graduation.

Student interns and graduates benefit from concurrent internship programmes in other ways. Final-year undergraduate students need to seek either employment or postgraduate education as the next step for their life development; concurrent internship programmes provide students with a way into the industry and a stronger outlook compared to other candidates in the eyes of human resource professionals [53]. Researchers [54] indicate that employers in the health profession tend to hire student interns after their internship programmes since they have gained an understanding of the working environment and have become a part of it. Therefore, internship programmes in the field of health science continue to be one of the best starting points for undergraduate students to gain on-site experience before graduation.

The duration of the internship is another point for discussion. Researchers [55] demonstrate that in Canada, undergraduate radiology students are required to participate in a year-long internship programme (i.e. nine months) in the field of didactic radiology in order to gain basic clinical training experience. In other health science and social care programmes, year-long internship experiences are usually encouraged, since clinical and health-related experience requires more time for supervision and observation [56]. However, unlike other clinical programmes (i.e. radiology, nursing, and psychology), public health programmes in North America usually require students to complete a semester-long or summer-long internship programme, as holding a license is not a requirement for public health professionals [57].

The outcomes of internships are also vital for participants. Researcher [58] has examined a large nursing student work-study internship programme with five university-partners in California. The work-study interns are paid and work three shifts per week for a total of 240 hours in a semester. To gain a specialised training perspective, nursing interns are asked to observe clinical responsibilities with clinical instruction and competence, such as physical assessment, medication administration, and intravenous skills. Although administrative work is required, the internship mainly focuses on clinical experience. Researchers [59] suggested that health and social care internship programmes might have two main types: generalised and specialised. For generalised internship programmes, students are required to intern at all related functional departments within the health facilities. Therefore, students may receive a general understanding of the functions and responsibilities of the health facilities. As for the specialised internship programmes, students are required to intern at only a few functional departments for detailed and specialised training and practices. Therefore, students are trained for particular knowledge and skills for the functional parts of the health facilities [59]. In fact, no one internship is better than another as the sites and universities should have negotiated the aims and purposes of the sites and the curriculum, particularly in health science, nursing, counselling, and psychology [60].

### 2.5. Disadvantages of Undergraduate Internship Programmes

Notwithstanding the benefits described above, negative outcomes for internship programmes also exist. Researchers [61] show that in current health professions, demanding workloads and multi-tasking working environments could reduce the beneficial outcomes of internship programmes. For example, researchers [62] demonstrates that supervisors at internship sites are usually frontline and senior professionals who need to participate in patient-care activities. Although some sites establish training departments for their employees, frontline training and relationships between patients and health professionals can only be developed through frontline practice. Therefore, limited resources, excessive workloads, and multiple responsibilities may lead to unclear outcomes, confusion regarding the sense of belonging, and dissatisfaction for both students and universities.

Researcher [63] has pointed out that, unlike other professionals, health professionals cannot make mistakes and must always exercise serious concentration. When health professionals take on supervisory internship responsibilities, they may not be able to pay adequate attention to both their interns and their patients. Further, having an increased workload with their duties at the internship site on top of their university assignments, stronger pressure, and excessive responsibilities, the morale of student interns and their supervisors could drop significantly, highly impacting the outcomes of the programme.

In conclusion, students who have completed concurrent internship programmes during their final year of undergraduate education are not yet entry-level professionals. However, they are more experienced than fresh graduates without experience in the real world. In fact, most employers tend to extend internship contracts to full-time employment if they receive satisfactory reports from on-site supervisors. The completion of an internship has been and is still considered one of the most meaningful requirements for health science and other vocational-oriented degree seekers.

## 3. Methodology

A qualitative methodology using the tools of in-depth interviews and focus groups seemed the most appropriate for this study. Because the goal of this research is to collect the in-depth feedback and opinions of the participants of this one-year-long on-site internship training programme partnering a university with a regional hospital and health centre, asking the participants face-to-face questions could capture the desired first-hand information without any hindrance or potential misunderstanding from the questionnaires [64].

### 3.1. Participants and Data Collection

Two groups, with a total of 44 participants (N = 44), were invited to participate. It is worth noting that all of the participants agreed to participate in this study. First, 32 trainees who had already completed the one-year-long on-site internship training programme along with their undergraduate degree in public health and healthcare were invited. Of them, 25 decided to remain in the regional hospital and health centre of their internship after graduation. Second, 12 departmental supervisors who had guided the trainees were invited to participate in two focus group activities, with six members per group. Three of those supervisors worked in the health promotion and disease prevention department (HPDP), three worked in the population and environment department (PE), two worked in the health administration department (HA), two worked in the human resources, training and development department (HRTD), and two worked in the public communication and marketing department (PCM).

Based on a review of related literature and research questions, two separate interview protocols—one for trainees and one for departmental supervisors—were developed to explore the significant aspects of this research. First, the researcher interviewed each of the trainees individually in a semi-structured, one-on-one, face-to-face in-depth interview, with each interview lasting 45–60 min and being consistent with interviewing criteria [65]. Second, the researcher conducted two sessions of focus group activities. The supervisors’ departmental backgrounds were randomly selected. For the first session, two HPDP supervisors, one HA supervisor, one HRTD supervisor, one PE supervisor, and one PCM supervisor were chosen. For the second session, one HPDP supervisor, one HA supervisor, one HPDP supervisor, two PE supervisors, and one PCM supervisor were selected. Each of the focus group sessions lasted approximately 60–70 min, and all were consistent with the interviewing criteria [66]. Semi-structured interview questions were asked of and discussed by the participants. All of the conversations were digitally recorded, transcribed, and returned (i.e., for member checking) to the trainees for validation of the content [67]. Once the participants approved their transcripts, the data were analysed employing the MAXQDA v.11 (VERBI GmbH, Berlin, Germany) qualitative data analysis software.

### 3.2. Data Analysis 

Themes and patterns that arose during the individual interviews and focus group activities with trainees and departmental supervisors were independently identified. An inductive approach was employed for this study [68]. The inductive approach allows the researcher to understand the feedback and opinions about the on-site internship programme between a university and a partnered regional hospital and health centre in the mid-western rural region in the United States of America. The researcher followed a general inductive approach [68] to narrow the massive size transcripts into first-level themes by employing the open coding technique [66]. The general inductive approach indicated that the data should be reduced even further [68]. Therefore, axial coding was employed to reduce the data into a second-level theme. The above procedures were conducted twice, as the data from trainees and departmental supervisors were independently separated [64].

### 3.3. Participants Recruitment and Research Site Access

This study involved two sites, a university and a partnered regional hospital and health centre in the mid-western rural region in the United States of America. Two groups, with a total of 44 participants (N = 44), were invited to participate. First, 32 trainees who had already completed the one-year-long on-site internship training programme along with their undergraduate degree in public health and healthcare were invited. Second, 12 departmental supervisors who had guided the trainees were invited.

The researcher contacted the potential participants through a printed letter to invite them to participate. As the researcher did not have any connections between the sites, the researcher sent the invitation letter for research with an invitation for participation to each individual (i.e., students and supervisors). The letter provided information including the rationale and purpose of the study, the nature of the data collection process, a declaration about their voluntary participation or non-participation, the protection of privacy, and risk. Within one week, the director of the regional hospital and health centre expressed the interests of this study. As a result, 44 participants agreed to join this study.

### 3.4. Protection of Subjects

The protection of human subjects is important to this research study, particularly for health science projects. One main concern is the protection of the participants’ identities. Therefore, the researcher made every effort to protect the identities of all participants and the research sites by masking their names. Protecting participant identities allowed all the participants to remain anonymous to any potential employers in their field. Also, the identities of both the university and regional hospital and health centre were masked.

### 3.5. Ethical Considerations

All potential participants, related personnel, and sites received written, and oral description of the project including its aims and expected outcomes and were informed of their rights to withdraw at any time without prejudice. Woosong University research department approved this study. The project was supported by Woosong University (2018-2019/WLI).

## 4. Findings

Although the goals of this one-year-long internship programme are to provide appropriate training to undergraduate students and skilled human resource power to the demanding public health and healthcare, most of the trainees and departmental supervisors expressed negative feedback and opinions regarding this internship programme.

Unlike the one-year-long gap-year system, in which students do not need to study any coursework and course modules, the current one-year-long internship programme requires students to complete full-time coursework during evenings and weekends, and to work at least part-time simultaneously. Currently, in the United States, most undergraduate programmes only require students to have 10–20 weekly internship hours in a workplace for a maximum of two semesters. Also, students are not required to go to the internship workplace during school breaks and national holidays. Therefore, the current one-year-long on-site internship training programme is one of the first of its kind in the profession.

Data were collected from (1) 32 trainees who had already completed the one-year-long on-site internship training programme, along with their undergraduate degree in hospitality and tourism management, and (2) 12 departmental supervisors who had guided the trainees. The researcher first reported the analysis of the data gathered from the 32 trainees from the on-site internship training programme. In the interviews, the trainees gave their feedback and opinions, discussing the advantages and disadvantages of the programme and their experiences.

### 4.1. The Perspective of the Trainees 

One of the outcomes of this internship programme is to provide the essential practical knowledge, soft skills, and the understanding of frontline arrangements to undergraduate students in the field of public health. Also, the feature of this internship programme is to provide generalised perspectives and training to future public health professionals. Therefore, trainees are required to intern in all functional departments in the regional hospital and health centre. The on-site internship programme, however, has its own positive and negative directions. The following parts outline the positive feedback and negative feedback from the perspectives of trainers and trainees.

In the interviews with the trainees, the researcher gathered both positive and negative feedback and opinions. Positive opinions mainly focused on the transferrable skills from textbooks to the workplace environment and the hands-on experience with frontline responsibilities. However, a large number of trainees expressed unforeseen career developments and promotions after the completion of the programme. The researcher determined six themes with which to categorise the findings of the trainees.

#### 4.1.1. Positive Feedback: The Transferrable, Practical, Frontline Experience 

One of the most significant themes from the data revealed by the trainees’ feedback was that of transferrable, practical, frontline experience. While the trainees were absorbing the practical skills from their internship experience, all of them needed to go through each department in the regional hospital and health centre for their holistic training. All 32 trainees indicated that the completion of the internship programme allowed them to exercise part of their textbook knowledge in their workplace. Trainee #32 described the experience at the health administration department as a “transferrable experience to the practice.” Trainee #27 reported that culinary arts could not be learnt from textbooks, just in kitchens, and stated that the “Textbook teaches us the nature of each communication, hospital allow[s] us to use the skills.” Likewise, Trainee #3 had found that “without the long working hours at the health promotion department, I [would] not even know how to communicate with patients.” Moreover, Trainee #18 described the practical experience in the frontline as a “life-changing experience, from a student to the professional worker in public health and healthcare” In short, the trainees indicated that the internship experience provided them with the opportunities to transfer their textbook knowledge into hands-on experience. Such practical knowledge could not be learnt from the classroom environment.

#### 4.1.2. Positive Feedback: The Experience of Operations Management and Administrative Skills

One internship requirement is to work in each department within the regional hospital and health centre, in order to acquire a generalist perspective. A large number of trainees expressed that the intensive training and directed experience from each department increased their skills and opened their horizons from the textbook level to the practical level. Three participants, Trainee #4, Trainee #8, and Trainee #17, indicated that the generalist perspectives allowed them to experience all functions within the regional hospital and health centre. Trainee #4 said, “I understood the entire functions of both operation and administrative management.” Trainee #17, further expressing the ideas about the interconnections among departments, said, “health promotion and disease prevention, population and environment, and health administration are all interconnected with each other. Each department supports each other’s functions. Although they have similar functions, without the hands-on experience, I could not manage the functions smoothly.” In short, particularly in small-size and regional hospital and health centres in the rural communities, human resources and manpower are some of the most significant challenges. Professionals in such environments should be well-prepared for multiple functions. The trainees advocate that the generalist perspective and training provided them with the opportunities to work as the multi-functional professions in their field. Several of them further advocated the interconnections among departments as being the key successful elements of this programme.

#### 4.1.3. Positive Feedback: The Demands of Acquiring Vocational Knowledge

Unlike large-size medical centres usually divide and separate their departments on the basis of each department’s functions, rural and regional hospitals and health centres usually merge departments into one division. As Trainee #26 stated, “I worked in the human resources, training and development department. I needed to learn knowledge and practical skills from the staff [for] the payroll, recruitment, benefits and compensation, and staff orientation.” A similar idea was shared by Trainee #29, who said of the experience in the health promotion and disease prevention department, “…community health promotion specialists do not handle and touch any procedures of cancer and disease promotion…So the knowledge is different from each other…I am glad that I can learn both in the term…” It is worth noting that several of the trainees appreciated that the goals of this programme are to train the trainees to know all the functions within the departments in order to gain a generalist perspective. Learning generalist skills and knowledge could increase their managerial and communicative skills among all departments.

One significant point from the interviews related to the multitasking and multi-learning expectations from the departmental exchanges, which allowed trainees to engage with all of the subfunctions within a large department. Whereas the overall functions of the population and environment department are to investigate and exercise the illness throughout the rural county, multiple subtasks were among the most influential factors that professional population specialists were required to handle. Trainee #7 mentioned that the “…population and environment department has multiple functions in environmental testing, water sources, health investigation, air problems, the relationship between population and the environment…all our trainees needed to go through all these functions for the generalist training.” Another trainee, #8, also shared about his/her intern times in the health promotion and disease prevention department,

Although the health promotion and disease prevention department has multiple functions, all the medical professionals only handle their own responsibilities…we all need to learn the coordination between medical professionals and supporting staff…for all administrative tasks, community promotional tasks, patient relations, and even counselling…the learning demand is very high…higher than my expectations…but it is worth [the effort] to learn…

More specific learning demands that were expected of the trainees included the ability to perform all logistics functions—from ordering, to purchasing, to deliver. In addition, the trainees were expected to use all of the different computer systems and to learn how to be transferred across multiple IT systems from different departments simultaneously. Trainees cited that the specialisations of each individual department limited many inter-departmental exchanges and interactions for effective communication, but the internships and hands-on experience in all departments allowed them to understand the functions of each chain within the system. Trainee #10 stated, “At the end of the internship, everything had [been] experienced…all trainees are the generalists of the entire functions of the regional hospital and health centre.” Some trainees highlighted the value that they acquired in communication skills from their telephone negotiations in the purchasing department, because they needed to bargain with at least five external organisations for their inventories. In short, it was impressive for trainees to extend their operational skills and interpersonal interactions, and their multitasking skills, as some of the advantages and long-lasting benefits for themselves and the organisation.

#### 4.1.4. Negative Feedback: Excessive Requirement of Knowledge, Skills, and Expectations

Positive feedback and opinions from trainees praised the fact that the generalist perspective and training allowed them to expand their horizons, from the beginning as unskilled undergraduate students to ending as skilled professional workers in the public health and healthcare profession. However, negative feedback constituted nearly two-thirds of their interview transcripts.

The excessive workload, and the knowledge and skills that the trainees were expected to master within a short period of time, were the most challenging aspects of the internship. Although the training and perspectives stressed becoming a generalist, some trainees wanted to gain the specialised skills within one or two individual departments for their own further development. Trainee #20 described that the population and environment department had six different functions. However, trainees could stay in each sub-functional division for no more than two weeks, due to the generalist perspective training. As a result, no particular specific skills could be mastered, and one trainee said, “…when I [had] just [learnt] how to investigate the environmental problems during the summer and high-temperature period, I [had] to switch to the population division for rural elderly and ageing problems.” Trainee #15 used the word “thunder storming” to describe how the trainees experienced each function. In addition, some trainees commented that their rapid assignment changes might increase administrative costs because each department was forced to spend resources and time for individual training. Trainee #13 mentioned,

Even if I do not ask any questions…the supervisor does not teach me…the department still needs to reserve a computer, space, and some manpower for trainees to sit down…during some peak seasons, our co-workers could not even use the restroom…how can they handle us…

In terms of specialisation, several trainees also shared that the excessive demands regarding knowledge and expectations decreased their interest in absorbing new knowledge from a department. Trainee #9 shared his experience in the logistics division, under the health administration department: “[Although] making a purchasing order needs to take at least months to master…trainees are required to make an order in one week…then make the bargaining purchasing in the following week…inventory in another week…” Trainee #14 further added her feelings about the preference for specialisation in some skills, sharing, “Making too [many] purchasing orders within a month…I [had] to learn [to] use the IT system, remember the name of the companies, the order of the forms…those are medical materials…I cannot make any mistakes…” The extraordinary degree of required skills and responsibilities increased the trainees’ skills and knowledge and their understanding of the daily functions of each department. However, the excessive workload and unmanageable knowledge requirements decreased the trainees’ effectiveness, and that reduction could be a negative outcome in their adult learning development and interest. In short, some trainees advocated that the training of individual departments should be extended in order to absorb in-depth understanding and functions of the division. It is worth noting that some trainees had shared the ideas about staying in an individual department for training. However, due to the generalised perspective and training direction, the trainees needed to transfer to the next department for further training.

#### 4.1.5. Negative Feedback: Unsureness about Career Opportunities and Promotions 

One goal of this one-year-long on-site internship training programme is to provide graduates with career opportunities upon graduation and at the same time to respond to the human resource shortages in the public health and healthcare profession. However, more than half of the trainees gave negative feedback about their further career development in the same regional hospital and health centre after graduation. The one-year-long work experience at the same regional hospital and health centre caused some trainees to consider switching their working environment and even to change their career pathway and development. Some trainees mentioned their desire to switch to another workplace after they had undergone intensive training in one regional hospital and health centre. Trainee #1 commented that the “…one-year contract is fair enough…I want to seek a better place outside of this interned location…” Others also complained that they could not acquire the necessary specialised skills and a sense of belonging because the switching occurred so often. Trainee #5 said, “I do not even know which department I belong to…what particular skills did I learn…” It is noteworthy that some trainees decided to change their career pathway after this intensive training programme.

Contrary to increasing the trainees’ professional skills and sense of belonging in the public health and healthcare profession, many felt that their generalist training eliminated their interest in learning and staying in one particular department within the public health and healthcare profession. Several trainees mentioned that the departmental directors and leadership limited their opportunities to be promoted as team leaders and supervisors because they did not gain the specialised skills in one particular department. Trainee #10 stated, “I [was] required to stay in the health promotion and disease prevention department, as I like…promotion of health behaviours…I requested from the university and the regional hospital and health centre…I was forced to switch to the others, as I have to follow the agreement…” Trainee #11 further described the limitations to building specialised skills and a sense of belonging in one particular department, stating,

I want to learn more about purchasing skills and procedures…when I started to read the invoice and payments…I was asked to go to health promotion…I want[ed] to build up the skills from the purchasing process, but I [had] to leave…When I asked to come back to the purchasing division after [my] completion of the training programme, the co-worker [one year ago] who sat next to me [had] been promoted as supervisor…but I still need[ed] to work as the entry-level staff…I felt that is unfair…

When they were busy gaining generalist and professional skills in each department by learning multiple tasks according to the holistic functions of the public health and healthcare, the trainees could not build the particular skills that are required in their preferred department, and more importantly, they could not achieve a sense of belonging in any of the departments. Significantly, some trainees also indicated that others who lacked a generalist perspective but who had particular skills were promoted even faster than they were. A sense of disappointment highly impacted their decision to leave the organisation and even the industry.

#### 4.1.6. Negative Feedback: Hardship of Work-Study Balance

One unique feature of this on-site internship training programme allowed students to complete their last year of university coursework while working at least half-time at the regional hospital and health centre. Some trainees indicated that the difficult balance of work and study impacted their performance in both their academic work and their professional accomplishments at work. Because the public health and healthcare profession runs 24 hours per day, the majority of trainees had never encountered such a scheduling challenge, and therefore they “[could not] even sit and listen to my instructors after 9 hours of working,” according to Trainee #2. Trainee #15 gave additional, common feedback, such as, “I need to maintain a good GPA and excellent performance at the intern centre…I really do not know how I can manage both…” As a result, a significant number of trainees decided to drop out of this internship programme and even abandoned their undergraduate degree due to the unexpected workload. In some cases, trainees were able to withdraw from the on-site internship training programme, but that decision could result in a failing grade that would impact their overall GPA.

In terms of academic performance, trainees need to receive evaluations of both their university coursework and workplace performance. In addition to their internships, trainees have to take theoretical coursework and lectures at the university. Most of the academic examination periods are either during or after the peak seasons of the year (e.g., Christmas, Labour Day, the summer holiday, etc.), so, as Trainee #12 mentioned, “The exams were scheduled after the Christmas break, how can I study as I have to work extensively over the New Year Holiday?” Because the examination periods and exam papers are standardised, a number of trainees may have had difficulty arranging such issues. In short, unlike the gap year trainees and post-graduation trainees, concurrent interns and trainees need to study and work at the site at the same time. In fact, some trainees indicated that the balance between study and work is one of the biggest challenges during the internship, particularly for trainees who are seeking merit scholarships. Therefore, the consideration of work-life balance became one of the negative areas of feedback for this programme.

### 4.2. Perspectives of the Departmental Supervisors 

By listening to the trainees’ feedback and opinions, the researcher identified several positive and negative themes associated with the one-year-long on-site internship training programme. Our findings are consistent with those of Knowles, who advocated that adults tend to absorb knowledge based on their vocational needs. In order to understand the holistic experience of this training programme, in the second stage of the data analysis, the researcher invited 12 departmental supervisors to participate who had mentored this one-year on-site internship training programme and the trainees. Whereas one of the goals of the internship programme is to provide potential manpower and respond to the immediate shortages in the departments, the departmental supervisors shared and echoed similar ideas and thinking during the focus group activities. The researcher identified three themes with which to categorise the findings of the departmental supervisors. Before discussing their reporting, it is worthwhile to note that after the data analysis, the researcher observed that more than two-thirds of the transcripts and sharing criticised different aspects of the nature of this one-year on-site internship training programme. The following section will outline the positive and negative perspectives obtained from the departmental supervisors.

#### 4.2.1. Positive Feedback: The Training Experience for Both the Trainees and Trainers

Our recording of the data according to the transcripts of departmental supervisors revealed that the supervisors felt the internship programme allowed them to improve and grow together with the trainees. This finding indicated that all 12 supervisors believed that both they and their trainees had gained knowledge from the one-year-long on-site internship training programme, under the intensive scheme. In the training experience, supervisors were required to guide the trainees, assuring that they learned the practical skills and operational steps in each department, learned the organisational structures with their guidance, met the goals of the training programme, and achieved the expectations of the organisation and the university. Although the trainees gained knowledge from the supervisors, the supervisors also absorbed and refreshed their own daily operations because of the trainees. Supervisor #3, from the population and environment department, said, “Guiding trainees from university allowed me to refresh my theoretical knowledge that I missed.” Supervisor #6, from the health promotion and disease prevention department, also echoed this idea, commenting that “…although the department has its own processes…the health promotion teams have refreshed the skills from our trainees…”

In addition to the operational management experience and knowledge they gained from the trainees, almost all of the supervisors argued that under the one-year-long on-site internship training programme, they could learn managerial skills, and sharpen leadership and mentorship skills, from the programmes and trainees. This finding is consistent with several emergent themes from the findings of the trainees, in which supervisors stressed the engagements of positive relationships and learning experiences. Further details of the data transcripts revealed that some supervisors indicated they earned promotions as senior supervisors due to the trainees’ positive feedback and their own mentorship experience from this one-year-long on-site internship training programme. Supervisor #9, from the population and environment department, said, “My trainees taught me public speaking skills from the textbook knowledge…we gained knowledge from each other…” Also, Supervisor #12, from the human resources, training and development department, advocated that some trainees provided knowledge of the contemporary HR system training to the entire department, saying that “…we learnt from each other…not just they learnt…” In short, a number of supervisors indicated that the supervising and mentoring might refresh their understanding of contemporary public health theories and knowledge due to the peer-sharing with their trainees. Furthermore, some supervisors also believed the internship experience allowed them to upgrade themselves as better leaders in the field. All functional supervisors advocated the benefits of this internship programmes does upgrade not only the knowledge of trainees but also the supervisors and related co-workers in the offices.

#### 4.2.2. Negative Feedback: The Short Duration of Specialised Training 

Although positive feedback and opinions were shared during the focus group activities, more than half of the feedback was negative. Two themes were identified that outlined the issues of this one-year on-site internship training programme. These themes echoed with one of the themes from the trainees’ speeches, in which most of the trainees complained about the excessive knowledge, skills, and expectations required of them within a short period of time. Unlike other internship programmes, in which trainees usually stay in no more than two departments and have specialised training, trainees in this programme needed to intern in each subdivision in the regional hospital and health centre to meet the generalist training schemes. In other words, each trainee could stay in the same department for no more than two months. Supervisor #1, from the health administration department, indicated that most of the trainees took two weeks to understand the steps of the department, another two weeks to learn the system, and another two weeks to master the processes. No further training could be provided, because the time was gone.

Three of the supervisors from the health promotion and disease prevention department indicated that operational management and skills in the hospital could be complicated. As Supervisor #5 shared, “How to prepare materials, teach the materials, and prepare for materials…it is impossible to teach them in two months…” Supervisor #6 echoed those ideas, saying that “…there are four K-12 schools in these rural county…we have to go to all these rural schools for health promotion…with four different types of students…no particular and detailed skills could be taught, as they have to go through all these schools in two months…” In short, based on the data from the supervisors’ transcripts, it is worthwhile to note that most of the supervisors could not teach and contribute specialised training to each trainee because the generalist training goals limited their mastery of detailed information.

#### 4.2.3. Negative Feedback: Lack of Stable Schedule 

Almost all of the departments in a regional hospital and health centre run at least 12 hours per day, and that schedule required both supervisors and trainees to work the day shift and also the overnight shift. Several supervisors indicated that due to the evening coursework schedules of the university, some trainees could not experience the evening operations. In addition to that sharing from the frontline departments, supervisors from the public communication and marketing department also spoke of similar difficulties. Supervisor #2 from public communication and marketing indicated problems for the overnight-shift learning experience, saying that “…the knowledge of disaster contribution and communication could be effective for the overnight shift…in fact, evening and weekend disaster happen…but not often…[there is] not much I can teach to the trainees besides the reporting…” Supervisor #7, from the health promotion and disease prevention department, indicated that the overnight shift schedule could not contribute, saying that “…our busiest time could be Saturday afternoon as some children may come during the weekend…but we cannot schedule our trainees, as many of them needed to go to Saturday evening classes…” In short, it is noteworthy that the schedules of university lectures limited the learning opportunities in several departments because of the peak hours in the regional hospital and health centre conflicted with the lecture hours of the university. Although the overnight shift is a favorite period for trainees and supervisors, most of the supervisors indicated that no particular knowledge could be taught during the non-peak hours.

## 5. Discussions

Although the findings for both trainees and departmental supervisors were reported in two separate sections, the data analysis for those two parts demonstrated alignment between the themes from both trainees and supervisors. According to the results of the study, both trainees and supervisors had already participated in and completed the one-year on-site internship training programme prior to the study. Both trainees and supervisors described the advantages and disadvantages of the one-year-long on-site internship training programme. However, more than half of the feedback and opinions indicated significant room for improvement.

According to the Theory of Adult Learning [21,22,23,24,25], adult learners tend to gain knowledge based on their prior knowledge and their needs for personal development. In addition, for the purpose of curriculum development and planning, curriculum, and internship programmes for adult learners tend to be practical exercise and vocational training. In this study, for example, the one-year-long on-site internship programme with the generalised focus matched and reflected with the ideas of the factors of adult learning planning [27]. For example, a number of supervisors and trainees stated that the one-year-long internship programme provided opportunities for both parties to gain the goals and objectives which reflected the daily practices [28].

In the trainees’ positive feedback and opinions, they highlighted the benefits of their transferrable, practical frontline experience, their experience of operations management and administrative skills, and the demands of acquiring vocational knowledge [33]. Consequently, it appears that the experiences of the one-year-long on-site internship training programme served as an early entry step for trainees because they helped them to attain important practical skills and working experience. Trainees also stressed that issues regarding the demands of acquiring vocational knowledge and skills were higher than their expectations [2], including the internships’ tasks in the health promotion, population and environment problems, health administration, and so on. Several trainees asserted that the generalist perspectives and schemes opened their horizons to all the essential functions that would be required of them as public health and healthcare professionals which echoed how adults gain knowledge from each other [25]. It is worth noting that according to the Theory of Adult Learning [21,22,23,24,25], sharing and reflecting from each other are the key successful elements for adult education. This study significantly enhances the communication bridge and the soft skills that are required in the field of health science.

The trainees also offered various negative feedback and opinions of the one-year-long on-site internship training programme, such as the excessive requirements in terms of knowledge, skills, and expectations, the unsureness about career opportunities and promotions, and the hardships of work-study balance [25,26]. As reviews of the outcomes of any programmes and schemes, the feedback and opinions from the participants are most important [5,14]. One highlight from the feedback and opinions was that the programme exceeded expectations. A prominent feature of this one-year-long on-site internship training programme is that trainees are required to intern in each department for approximately one to two months. However, the functions of each department can be unique. Most expressed that parts of the training were missing from the departments. As Trainee #6 shared, “I wish I [could] learn more about kindergarten and secondary school health promotions, but time [flew].” Several of the trainees indicated that they were interested in staying in a department for the subsequent internship months, but based on the internship agreement, the trainees had to move on to the next department. In fact, the desire for learning in one particular department was often mentioned. The current internship programme has provided the most comprehensive training and arrangements for all parties thus far [52,55,56]. Currently, most of the internship programmes in the field of health science tend to be semester-long or summer-only programmes. It is worthwhile to note that flexibilities should be introduced in order to respond to the feedback from participants in the programme (i.e., from both supervisors and trainees). Currently, both parties stated that the limitations of the selections and scheduling inhibited the programme’s effectiveness.

In addition to the trainees, the frontline departmental supervisors were also essential participants in this one-year-long on-site internship training programme. Although the supervisors’ feedback was limited due to the nature of focus group activities, many of them continued to express sensitive ideas with others. In terms of positive feedback and opinions from the supervisors, the only positive feedback was about the training experience for both trainees and trainers. In that category, nearly all supervisors argued that they viewed the one-year long on-site internship training programme favourably, as it helped them to gain managerial skills for their promotions [28]. Such positive feedback was reflected in the literature about how hospital-based student internship programmes between the university and other 19 community-based hospitals successfully placed prospective health professionals into the right positions after university graduation [52]. Supervisors also stressed the benefits of refreshing their skills and knowledge through the trainees and the fact that that allowed them to plan effectively [13].

Another finding that has the potential to influence the curricular reform is the duration of the internship programmes for rural communities, sites, and populations [62]. It is worth to note that although some negative opinions were shared. This one-year-long internship programme provided an alternative option for prospective health professionals to understand the working conditions and environments in the field. The current internship programme is one of the first one-year-long internships in the region as most of the current health science internship programmes tend to be semester-long or summer-only programmes [61,62]. Although the current feedback of the one-year-long internship programme received both positive and negative feedbacks, further developments, enhancements, and improvements may be implemented. On the other hands, from the literature review, semester-long or summer-only internship programmes received the same amount of negative opinions from both supervisors and trainees due to the duration issues [63]. Therefore, the current internship programme, however, provided longer training which assisted the prospective health science professionals in establishing a sense of belonging and the essential skills that are required for the current workplace [49].

However, and most importantly, more than half of the departmental supervisors gave negative feedback and opinions with regard to the short duration of specialised training and the lack of stable schedules. One highlight from these two themes was the two-month-long requirement. Supervisor #8, from the public communication and marketing department, indicated that “connecting the government department, state department, and community leaders could not be learnt in two months…an appropriate license and skills are required…the two-month long training…is good for the administrative part only, but [not] for the practical skills…” It is important to note also that most of the supervisors were concerned about the duration of the *internship* because they did not understand how to contribute particular practical skills and knowledge in a programme that differed so radically from traditional internship programmes that have at least six months of specific training. Due to the feature of the programme’s generalist perspectives, the supervisors felt that the maximum outcomes of this generalist training were limited [26].

In conclusion, a number of supervisors and trainees advocated the two-month long duration at each functional department is one of the challenges of the current internship programme. Such a limited duration of internships for each functional department with negative feedback does not only occur in this current one-year-long internship programme. In fact, previous literature indicated that many of the health science internship programmes only lasted for a semester [48,49,50,51,52]. Students could not receive essential training and build up a sense of belonging within a short period of time (i.e. two-month or a semester long). However, the current one-year-long internship programme provided the opportunities to establish a sense of belonging and understanding of the sites and rural communities with mentoring in the site. Further, although the trainees received individual training from each functional department, the practical experiences would not limit the trainees’ experiences and knowledge acquisition.

### Practical Implications

First, regarding an internship programme that allows undergraduate students to spend one year of their university voyage in the practical workplace, the feedback and opinions from the participants (i.e., the supervisors and trainees) should be combined in order to formulate a programme that fosters effective performance [25,26]. As additional, similar internship programmes with generalist perspectives and longer durations (e.g., one-year-long or two-year-long sessions) may be established due to the profession demands and responses from students, the current study’s indications about programme performance and its results will allow university administrators and public health and healthcare leaders to evaluate and plan effectively [10,11,14,18,20]. Once these one-year-long internship programmes become commonplace, the approach may be extended not only throughout the public health and healthcare profession, but also to other fields in general. In the meantime, given the features of the generalist perspectives of the one-year-long on-site internship training programme, the results have demonstrated that the trainees were prepared to work in all departments due to the extraordinary experiences they could gain from all of the functional parts of a regional hospital and health centre, and particularly from the experience of understanding the communication and healthcare administration processes [6].

Second, the application of the one-year on-site internship training programme also provides a blueprint for other undergraduate programmes to reform their internship programmes. Potential academic fields include but are not limited to nursing, social work, mental health, and counselling, all of which require workers to have generalist perspectives and specialised skills in the workplace [30].

Third, practical implications that this study offers to the public health and healthcare profession suggest that the placement of interned trainees after their completion of the programme will not only help to establish the programme’s attractiveness to potential trainees but also will immediately help solve the long-term employee shortage of appropriate employees [12]. Finally, supervisors gained numerous leadership and managerial skills that allowed them to be promoted as skilled managers and directors of their departments. As long-term internships become more prominent in the public health and healthcare profession, improvements, flexibilities, and arrangements should be incorporated.

Fourth, professional education and educational curriculum planning for health science should design the practice preferences of health professionals toward assisting rural and remote communities and populations [59,60,61,62,63]. Therefore, it is significant for colleges and universities to encourage and review the on-site internship training programmes. University department of health science, nursing schools, and social caring departments may consider designing one-year-long internship programmes and committed to delivering internship training programmes to rural and remote communities. As the literature review stated, most of the internship training programmes in the field of health science in the North American environment tended to be semester-long or summer-only programmes with a specialised focus. Such specialised focused programmes may only fit the needs of urban sites with individual departments. However, for rural sites with limited resources and manpower, generalised perspectives and long-term internship training programmes would better fit the actual situation.

Fifth, this study goes beyond the existing literature to explore the significance of the relationship between supervisors and trainees. In the current literature, researchers indicated that some of the current nursing internship programmes tend to be specialised-oriented programmes which nursing students may stay in no more than two departments for the training. However, for public health professionals in rural communities and sites, generalised-oriented programmes could fit the actual situation, particularly for the limited resources and the shortage of manpower.

Sixth, recommendations for international health science and public health internship and training programmes are also the main points for implementation in this study. In the current literature review about the internship programmes in international locations [57,58], a number of internship programmes in the field of health science and public health only require a semester-long, three-month-long, or summer-only training at the sites. The current research indicated that year-long training could increase the understanding, practical skills, and the sense of belonging of students. Researchers also indicated that in the current Indian public health environment, university students, however, require less than a year to complete internship programmes with. The project indicated that the limited duration of internship and training programmes provided the disadvantage of public health students as the limited duration of training may not allow students to gain the essential and overall understanding and skills in the particular field [69]. This reflects the current study about how a one-year-long internship programme with a generalised perspective could benefit students in the fields of health science and public health.

Seventh, a recent research project compared the public health and patient care internship programmes, particularly in the field of pharmacy, among India, USA, Finland, and Denmark. The project indicated that students at the Bachelor of Pharmacy programmes only require completing a three-month-long internship. Finnish students at the Bachelor of Pharmacy programmes only need to complete a six-month-long internship. Danish students at the Master of Science in Pharmacy programmes only require completing a six-month-long internship. Based on the research on this study, semester-long or six-month long internship programmes, however, may not allow trainees to establish the overall practical skills and sense of belonging towards the health facilities [70]. Therefore, the implementation of the current study provided an example and blueprint for other related health science programmes to reform their internship programmes at the site. For example, expanding the duration of the six-month-long internship programmes could help with responding to the demand of the current challenging health environment [53,54].

Eighth, in addition to the above cases in India and Europe, the current health science internship programmes in Nepal and other South Asian countries also face the problems of limited-duration internship training. The internship training in Nepal, however, only requires a two-week long community internship programme. The current study could provide assistance for the current health science professional and university administrators to reform their health science curriculum as generalised internship programmes, and its aims and goals could assist both students and the health facilities to secure the necessary manpower. Unlike the North American environment, Nepal and South Asian countries may have their own practice and professional training schemes. However, the outcomes of this study may provide the direction of curriculum reforms and the development of their current internship programmes to Asian universities and health facilities for manpower and training purposes [70,71].

## 6. Conclusions

First, each research must have its own limitation. This study has a number of limitations, associated with the participants involved, the sites, the locations, and the landscape of related research. Most importantly, this study is deliberately limited in scope and aim, and therefore it is not possible to outline conclusions about both national and international problems and situations. The current study indicated that relationship between the current generalised perspective of the internship programme, practical skills acquisition, and duration of the training could be beneficial for all parties (i.e. university department, students, and health facilities). However, it is possible that these particular outcomes were also related to North American situations with particularly challenging and difficulties. Without further and cross-boundary research and practice, it is not possible to indicate that the situations noted here are equally relevant outside of the North American environment.

Second, more research into internship performance and implementation in the public health and healthcare profession is needed in order for us to understand and evaluate the quality of internship programmes, regardless of their duration, features, and perspectives [15]. Future research into on-site internship training programme performance should be planned for different fields, not only from a public health and healthcare profession standpoint but also from the perspectives of other fields that require significant practical experience. In addition, because the generalist perspectives of this one-year-long on-site internship training programme are a key feature, other fields, such as those of nursing, counselling and social work, could adopt similar generalist perspectives in order to train generalists with interdepartmental skills. As the literature review indicated, currently, most of the North American internship programmes tend to be semester-only or summer-only programmes, professionals, university supervisors, and site administrators may use this study as a blueprint to implement tailor-made internship programmes which fit the needs of their professions [48,49,50,51].

Third, the one-year-long duration is uncommon in the current curricula in academia. In fact, some universities employ the gap-year system [38,39,40,41,42,43,44,45], which does not require students to take coursework on campus [4]. However, for the current programme, the feature of working as nearly full-time employees who receive frontline mentorships with departmental supervisors in the workplace increases the interns’ sense of belonging and allows both trainees and supervisors to establish positive engagements and relationships [12]. However, as the literature review indicated, one-year-long internship programmes in the field of pharmacy in Canada successfully assisted both trainees and the sites for human resources and soft skill training purposes [52,53,54]. Therefore, the university department and the partnered sites could discuss and redesign their duration of internship programmes due to the needs of their regions and populations. After the innovative internship programmes are created, researchers may further evaluate the results of the internship programmes.

Fourth and finally, the one-year-long on-site internship training programme could be coordinated with other local, mid-size and small-size public health and healthcare organizations in which human resource shortages are also significant [52,55]. If the current one-year-long on-site internship training programme can be completed in larger medical centres in suburban and urban regions, trainees could potentially do more in-depth training in each individual department and receive stronger mentorships.

All of the issues notwithstanding, the benefits and advantages of a one-year-long on-site internship training programme are successfully investigated in this study. Further experimental internships, and associated improvements will be welcome and should be conducted in other workplaces and in other fields.

## Figures and Tables

**Table 1:** Factors of adult learning planning.

Factors of Adult Learning Planning	
Preparing the learners	The curriculum and learning plan should be designed to match and engage with the prior life experience of the learners. It should assist the learners in engaging in activities that allow them to develop the skills to solve the problems.
Designing the environmental setting	The learning environment should be respectful, effective, informal, engaged, interactive, and supportive.
Engaging in planning	Each learner may contribute his or her unique prior knowledge and experience to the curriculum and learning plan. Do not ignore participation and sharing from any learners.
Identifying the needs of the acquisition	As the curriculum and learning plan become rich, learners can identify the needs for their own problems.
Establishing the goals and objectives	Goals and objectives should be flexible, changeable, and negotiable between instructors and learners, throughout the learning process, because the needs of learners differ from time to time.
Planning for learning through prior experience and interaction	Engage and encourage learners to share their prior experiences to solve problems using their new knowledge via interactive collaborations with other learners.
Identifying applicable activities	Employ inquiry projects, discussions, case studies, sharing, and experimental cases.
Evaluating performance	Performance can be qualitative and quantitative. Potential evaluations could be conducted through a collaboration among peers for a case study or problem.
